# Genome sequence of the entomopathogenic *Serratia entomophila* isolate 626 and characterisation of the species specific itaconate degradation pathway

**DOI:** 10.1186/s12864-022-08938-2

**Published:** 2022-10-27

**Authors:** Amy L. Vaughan, Eric Altermann, Travis R. Glare, Mark R. H. Hurst

**Affiliations:** 1 Bio-Protection Research Centre, Lincoln University, Lincoln, Christchurch, New Zealand; 2 AgResearch, Resilient Agriculture, Lincoln Research Centre, Christchurch, New Zealand; 3 AgResearch, Consumer Interface, Hopkirk Research Centre, Palmerston North, New Zealand; 4 Riddet Institute, Massey University, Palmerston North, New Zealand

**Keywords:** Horizontal gene transfer, Entomopathogen, Genome, Chromosome, Itaconate, Virulence

## Abstract

**Background:**

Isolates of *Serratia entomophila* and *S*. *proteamaculans* (Yersiniaceae) cause disease specific to the endemic New Zealand pasture pest, *Costelytra giveni* (Coleoptera: Scarabaeidae). Previous genomic profiling has shown that *S. entomophila* isolates appear to have conserved genomes and, where present, conserved plasmids. In the absence of *C. giveni* larvae, *S. entomophila* prevalence reduces in the soil over time, suggesting that *S. entomophila* has formed a host-specific relationship with *C. giveni*. To help define potential genetic mechanisms driving retention of the chronic disease of *S. entomophila*, the genome of the isolate 626 was sequenced, enabling the identification of unique chromosomal properties, and defining the gain/loss of accessory virulence factors relevant to pathogenicity to *C. giveni* larvae.

**Results:**

We report the complete sequence of *S. entomophila* isolate 626, a causal agent of amber disease in *C. giveni* larvae. The genome of *S. entomophila* 626 is 5,046,461 bp, with 59.1% G + C content and encoding 4,695 predicted CDS. Comparative analysis with five previously sequenced *Serratia* species, *S. proteamaculans* 336X, *S. marcescens* Db11, *S. nematodiphila* DH-S01, *S. grimesii* BXF1, and *S. ficaria* NBRC 102596, revealed a core of 1,165 genes shared. Further comparisons between *S. entomophila* 626 and *S. proteamaculans* 336X revealed fewer predicted phage-like regions and genomic islands in 626, suggesting less horizontally acquired genetic material.

Genomic analyses revealed the presence of a four-gene itaconate operon, sharing a similar gene order as the *Yersinia pestis ripABC* complex. Assessment of a constructed 626::RipC mutant revealed that the operon confer a possible metabolic advantage to *S. entomophila* in the initial stages of *C. giveni* infection.

**Conclusions:**

Evidence is presented where, relative to *S. proteamaculans* 336X, *S. entomophila* 626 encodes fewer genomic islands and phages, alluding to limited horizontal gene transfer in *S. entomophila*.

Bioassay assessments of a *S. entomophila*-mutant with a targeted mutation of the itaconate degradation region unique to this species, found the mutant to have a reduced capacity to replicate post challenge of the *C. giveni* larval host, implicating the itaconate operon in establishment within the host.

**Supplementary Information:**

The online version contains supplementary material available at 10.1186/s12864-022-08938-2.

## Background

The genus *Serratia* comprises ubiquitous species of obligate symbionts and opportunistic pathogens. Their success is, in part, due to their production of a wide range of proteases, lipases, and chitinases, as demonstrated in *S. marcescens* [1, 2], which have been implicated in the degradation of the insect exoskeletons and gut epithelial tissue [3, 4].

*Serratia entomophila* is the causal agent of amber disease and is used as a biopesticide in exotic grass pastures for control larvae of the endemic pest, *Costelytra giveni* (Coleoptera, Scarabaeidae) [5]. The main insect virulence determinants of *S. entomophila* are encoded on the amber disease-associated plasmid pADAP [6] which encodes two virulence factors, the Sep Toxin complex [7], and the Anti feeding prophage (Afp) (Hurst 2004). pADAP-bearing isolates of *S. entomophila* and some plasmid-bearing isolates of *Serratia proteamaculans* are implicated in a host-specific chronic infection *C*. *giveni* larvae that can take 2–3 months after ingestion of bacteria before larval death. Due to the weakening of the host intestine over time, the bacteria eventually gain entry to the haemocoel causing death by septicemia [8]. Recently, a highly virulent isolate (AGR96X) of *S. proteamaculans* with bioactivity towards both *C. giveni* and New Zealand manuka beetle *Pyronota spp*., has been identified that causes the death of larvae within 5–12 days of ingestion [9].

To date, only single isolations of *S. entomophila* from outside New Zealand, in France, Mexico, and India, have been reported [10–12]. Restriction enzyme profile assessment of the chromosomes and plasmids of *C*. *giveni* active strains revealed isolates of *S. entomophila* appeared to be genetically conserved while those of *S. proteamaculans* were more varied [13–15].

Unique to *S. entomophila* is its ability to utilize itaconate, which can be used to differentiate *S. entomophila* from other *Serratia* species [11, 16]. Recent research has highlighted the importance of itaconate as a eukaryote-derived antibacterial metabolite [17]. *Yersinia pestis* and *Pseudomonas aeruginosa* encode an itaconate degradation pathway enabling the bacteria to convert host-derived itaconate (methylenesuccinate) into pyruvate and acetyl-CoA, allowing the pathogen to survive [18]. The utilization of itaconate by *Y. pesti*s enables the bacterium to survive in host macrophages [18]. The cleavage of isocitrate into succinate in the glyoxylate cycle in the itaconate degradation pathway has been implicated in fungal and bacterial pathogen persistence [19].

Through use of itaconate selective medium as a basis to isolate *S. entomophila* from soil Jackson et al. [20] found that, in the presence of *C. giveni* larvae, the number of *S. entomophila* cells in soil increased from an average of 5 × 10^4^ CFU/g soil to as high as 10^7^ CFU/g soil with increasing larval density. This was followed by a rapid decline of *C. giveni* larvae and a subsequent decline in *S. entomophila* in soil samples to the point where the bacterium was no longer able to be isolated on selective media. This, combined with the host-specific nature of *S. entomophila* towards *C. giveni* and the chronic nature of amber disease, suggests that *S. entomophila* may be co-evolving with its host in a predator–prey relationship [20].

In this study, we describe the first reported genome sequence of *S. entomophila,* of isolate 626, the active agent of the commercial biopesticide BioShield® [21]. The ability of *S. entomophila* to degrade itaconate was also characterised. Through in silico analysis, we sought to define unique genetic adaptations of *S. entomophila* required for long term interaction in *C. giveni,* and its limited ability to survive long term in the soil.

## Results

### Annotation and overview of the *Serratia entomophila* 626 genome 

The *S. entomophila* 626 genome sequence comprises one complete chromosomal contig and a single plasmid contig, the latter previously annotated by Sitter et al. [15]. The *S. entomophila* isolate 626 chromosome comprised of 5,046,461 bp, of a comparable size to *S. grimesii* (5,072,299 bp) but smaller than *S. proteamaculans* (5,593,263 bp, Table 1). The G + C content of *S. entomophila* (59.1%) is similar to *S. nematodiphila*, *S. marcescens,* and *S. ficaria,* but ~ 5% higher than *S. proteamaculans* and *S. grimesii.* The chromosome of *S. entomophila* encodes 22 rRNA genes compared to 12 *S. grimesii* rRNA genes, but similar number to the 22–24 rRNA genes noted in the other assessed *Serratia* species (Table 1).

**Table 1:** Genome statistics of individual *Serratia* spp. isolates assessed in the study

*Feature*	*S. entomophila* 626	*S. proteamaculans* 336X	*S. marcescens* Db11	*S. ficaria* NBRC 102596	*S. grimesii* BXF1	*S. nematodiphila* DH-S01
Chromosome size	5,046,461	5,593,263	5,113,802	5,261,721	5,072,299	5,224,920
GC content (%)	59.1	54.9	59.5	60.1	52.8	59.5
CDS	4,695	5,138	4,848	4,896	4,787	4,789
tRNA	80	92	87	86	78	90
rRNA	22	22	22	22	12	24
Host	*Costelytra giveni*	Wheat	*Homo sapien*	*Homo sapien*	*Bursaphelenchus xylophilus*	*Heterorhabditidoides chongmingensis*
GenBank Accession number	CP074347	NZ_CP045913.1	NZ_HG326223.1	NZ_BCTS00000000.1	LT883155	NZ_CP038662

**Fig. 1:**
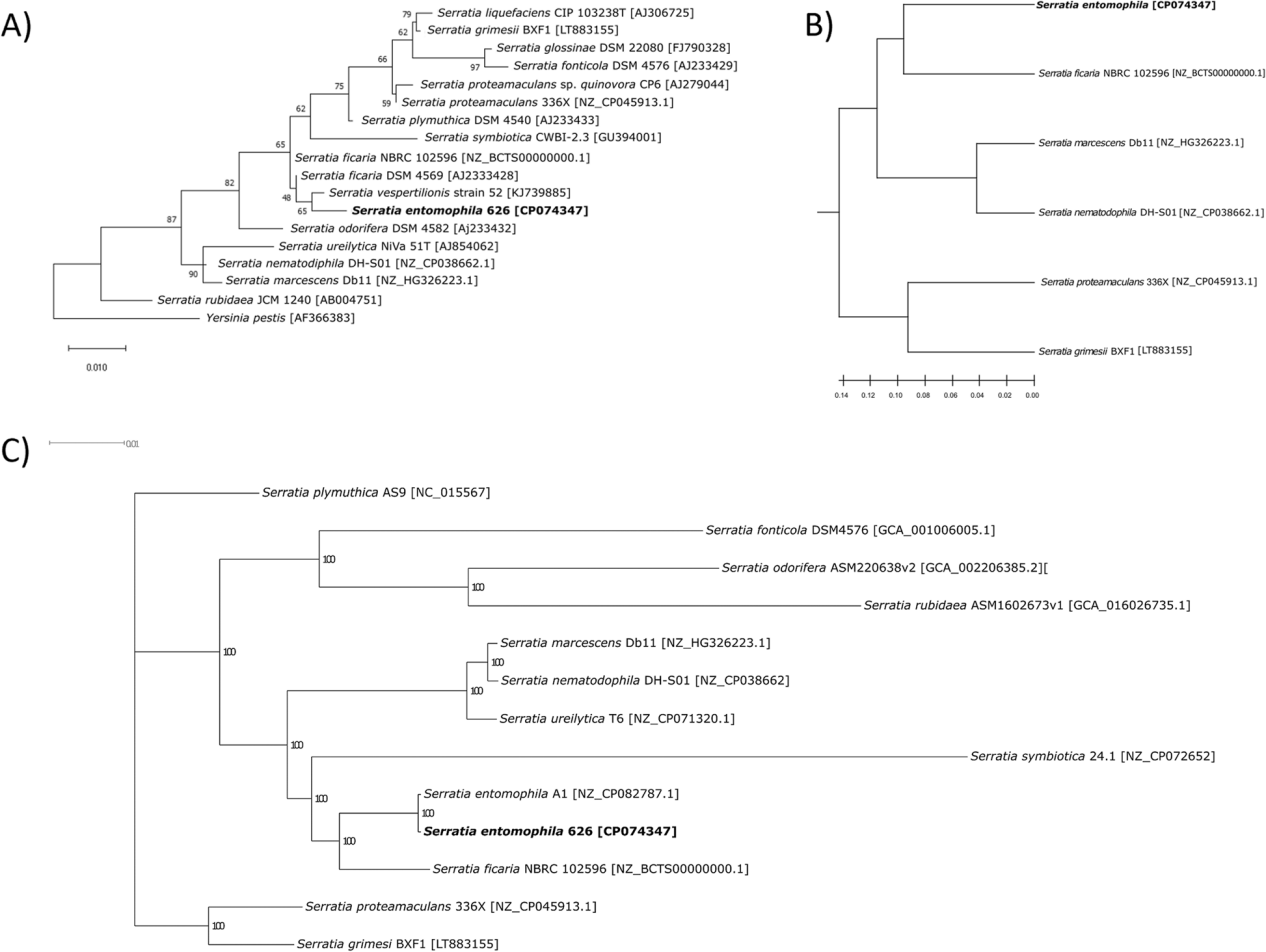
Inferred phylogeny of *Serratia entomophila* within the *Serratia* genus. **A** 16S rDNA Maximum likelihood tree of 18 sequenced *Serratia* spp. Percentage of trees shown in which the associated taxa cluster together is shown next to the branches. Branch lengths measured in the number of substitutions per site. This analysis assessed 17 members of the *Serratia* genus with *Yersinia pestis* used as an outgroup. Accession numbers for each 16S sequenced used is shown in square brackets. *Serratia entomophila* 626 is indicated in bold. **B** Functional genome distribution (FGD) analysis of representative complete *Serratia* genomes. The predicted ORFeomes of all 6 genomes were subjected to an FGD analysis [24], and the resulting distance matrix was imported into MEGA11 [25]. The functional distribution was visualized using the UPGMA method [26]. **C** Nonrecombinant core phylogeny of *S. entomophila* 626 and 12 representatives of the *Serratia* genus including the *S. entomophila* A1 type strain

Based on 16S rDNA phylogeny, *S. entomophila* shares the highest similarity to *S. vespertilionis* and *S. ficaria* (Fig. 1A). Functional genome distribution (FGD) analysis of the selected *Serratia* species assessed in this study found *S. entomophila* was most similar to *S. ficaria* (Fig. 1B) which was corroborated by nonrecombinant core genome phylogeny. wherein the *S. entomophila* A1 type strain is included (Fig. 1C). The *S. entomophila* type strain A1 and isolate 626 share 99.4% nucleotide identity as determined by LastZ chromosomal alignment in Geneious version 10.2.6 [22, 23]. Core phylogeny was built on a concatenated alignment of 630 nonrecombinant single copy gene groups, resulting in strong support of tree nodes and correlating with the 16S and FGD analysis.

**Fig. 2:**
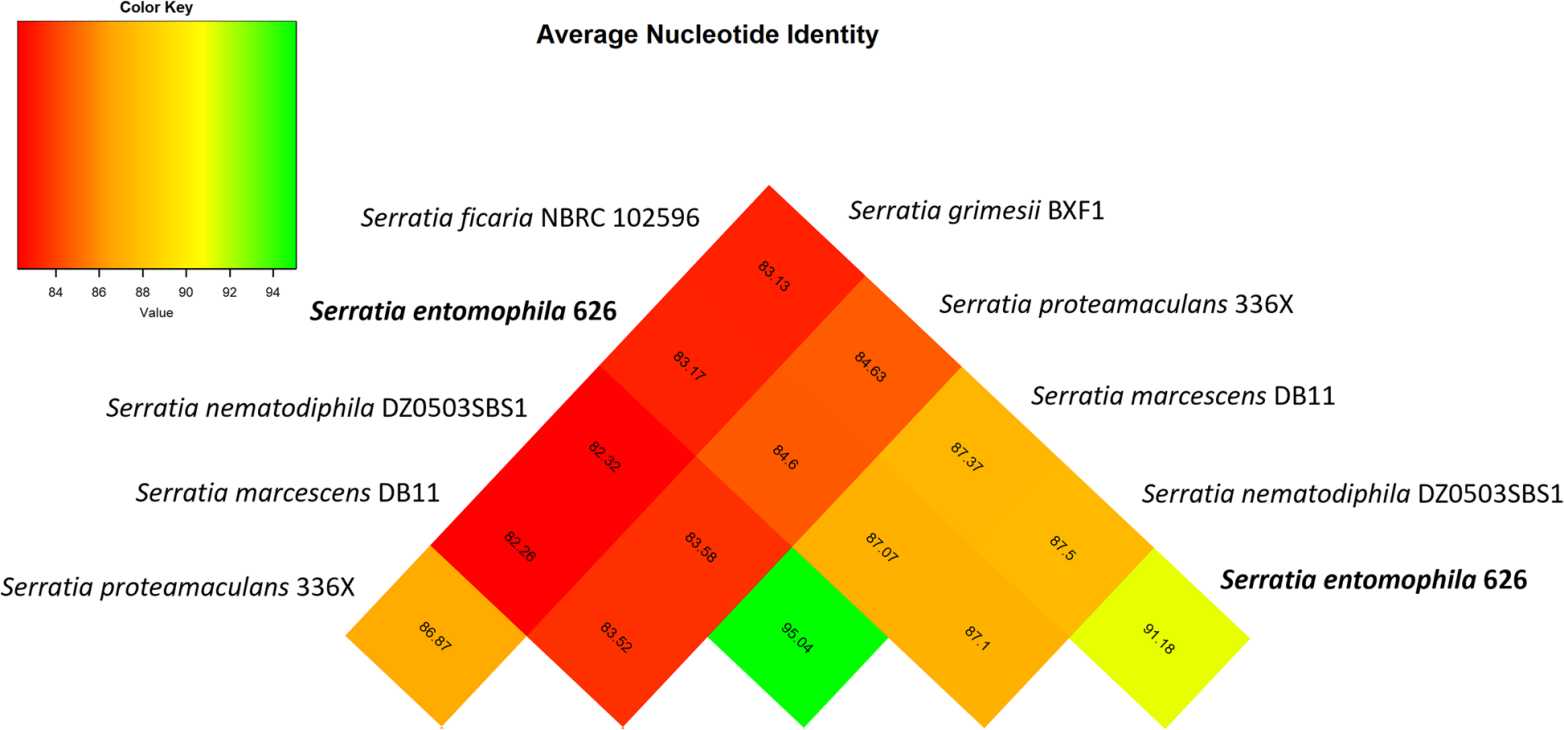
ANI values of all the comparative *Serratia* isolates used in this study displayed in a heat map derived from the comparative matrix. Green denotes nucleotide > 95% percentage similarity, red to yellow reflects lower nucleotide similarity values. *Serratia entomophila* 626 shown in bold

To further define the relatedness of *S. entomophila* to the selected *Serratia* species, the genomes of the strains were assessed by ANI. *S. entomophila* isolate 626 shared highest ANI of 91.2% with *S. ficaria* followed by *S. marcescens* and *S. nematodiphila* at 86% with *S. proteamaculans* 336X only sharing 84.6% nucleotide identity (Fig. 2). *S. vespertilionis* is now considered a heterotypic synonym of *S. ficaria*, with 99.5% sequence similarity between the type strains [27].

**Fig. 3:**
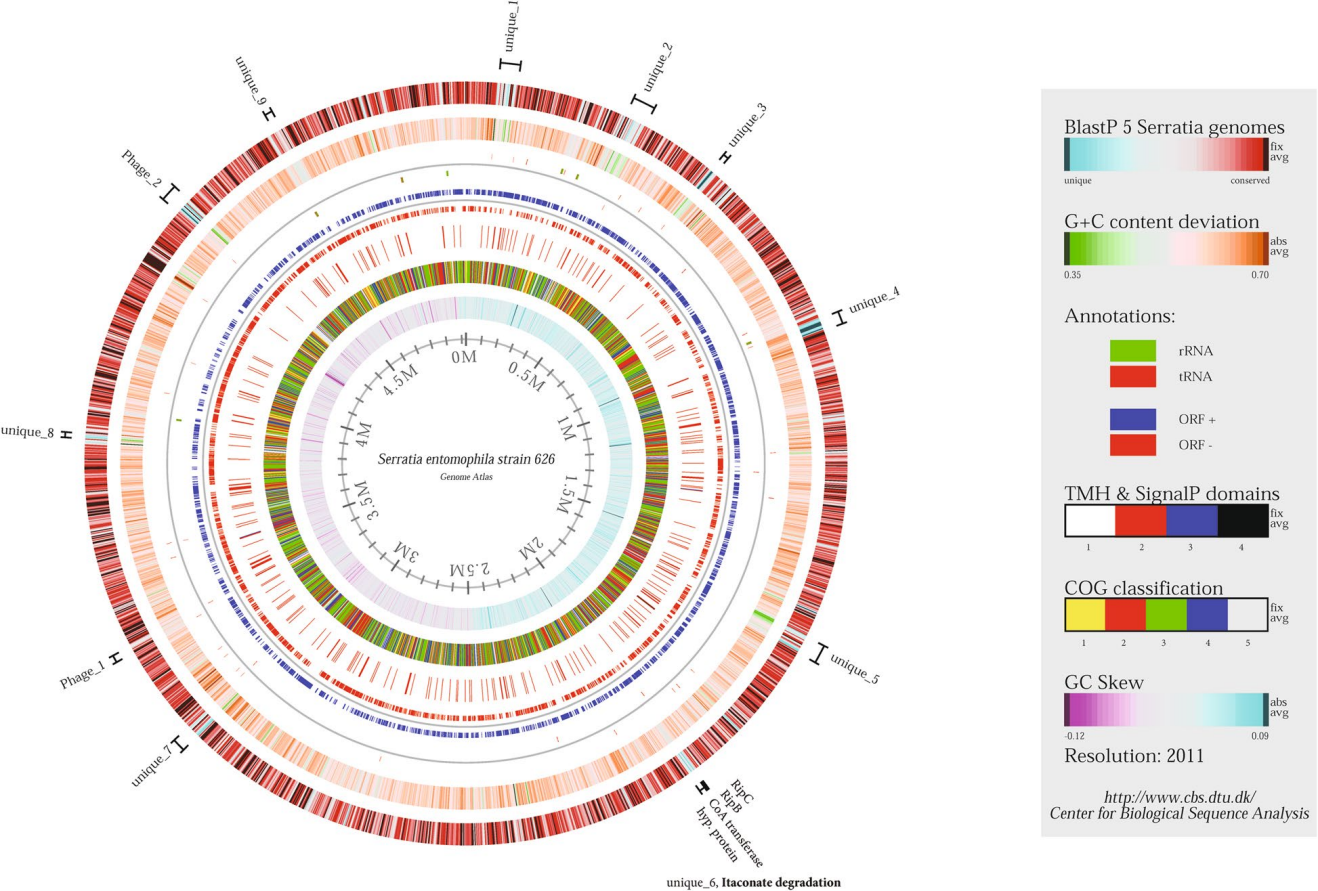
Genome atlas for *Serratia entomophila* 626. The genome atlas outermost circle shows BlastP similarities against the five *Serratia* isolates assessed in the study. Regions in blue represent unique proteins whereas red indicates high levels of conservation. Inner circle 2 shows GC content deviation, where dips below the average GC content are shown in green, and high spikes in orange. Circle 3 shows annotations of rRNA (Green) and tRNAs (Red) encoded on the forward and reverse strand. Circle 4 shows ORF orientation either in sense (+, Red) or antisense (-, Blue) orientation. Circle 5 shows the prediction of Signal peptide domains. Outer circle 6 shows assigned COG classification assigned into categories 1–5, 1) Information storage processing 2) cellular processes and signalling 3) metabolism 4) poor characterisation 5) uncharacterised or no assignment. The final innermost circle shows GC skew. Unique regions and phages are highlighted and numbered. Phage_1 denotes the DinI encoding phage. Unique_6 denotes position of the Itaconate degradation operon

Alignment and BlastP vs BlastP analysis of the *S. entomophila* genome against the selected *Serratia* species, identified eleven large *S. entomophila* unique regions (Fig. 3). Two of these regions coincided with phage elements. Unique region 6 encoded genes associated with itaconate degradation, a property specific to this species.

**Fig. 4:**
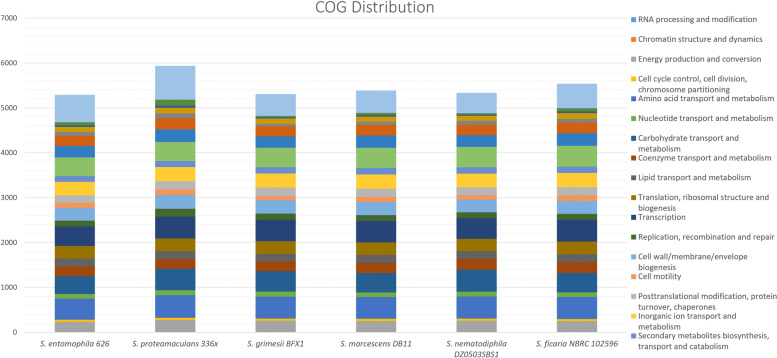
Distribution of COG functional categories for *Serratia* spp. Percentage COG distributions of annotated genes and their functions in the complete chromosomes of species belonging to the *Serratia* genus. The cumulative stacked count shown for each species representative. Full COG breakdowns listed in Additional File 1

Reflecting chromosome size, *S. entomophila* 626 encodes fewer predicted proteins (*n* = 5,289) than *S. proteamaculans* 336X (*n* = 5,935) (Table 1). Figure 4 presents the clusters of orthologous groups (COGs) of the assessed *Serratia* (detailed in Additional File 1). Irrespective of genome size the percentage genome allocation to these COG clusters reveals key differences. *S. entomophila* 626 features a noticeable reduction in proteins assigned to energy production and conversion when compared to other *Serratia* isolates (Category C). Relative to *S. proteamaculans* isolate 336X, 626 encodes fewer phage-associated proteins and replication (Category X), while 626 encodes more COGs assigned to translation, ribosomal structure, and biogenesis (Category J). Across the assessed *Serratia* species, there were no noted differences in either the cell defence (Category V) or secondary metabolites biosynthesis, transport, and catabolism (Category Q). The *S. ficaria* isolate NBRC 102596 encodes more energy-producing (+ 0.52%) and carbohydrate metabolism (+ 1.55%) genes than *S. entomophila* 626 (Category C, 4.48% and Category G, 7.45% respectively). Relative to *S. ficaria, S. entomophila* 626 encodes for 0.3% more phage-derived proteins (Category X) in addition to 0.14% more allocation to cell defence mechanisms (Category V).

**Fig. 5:**
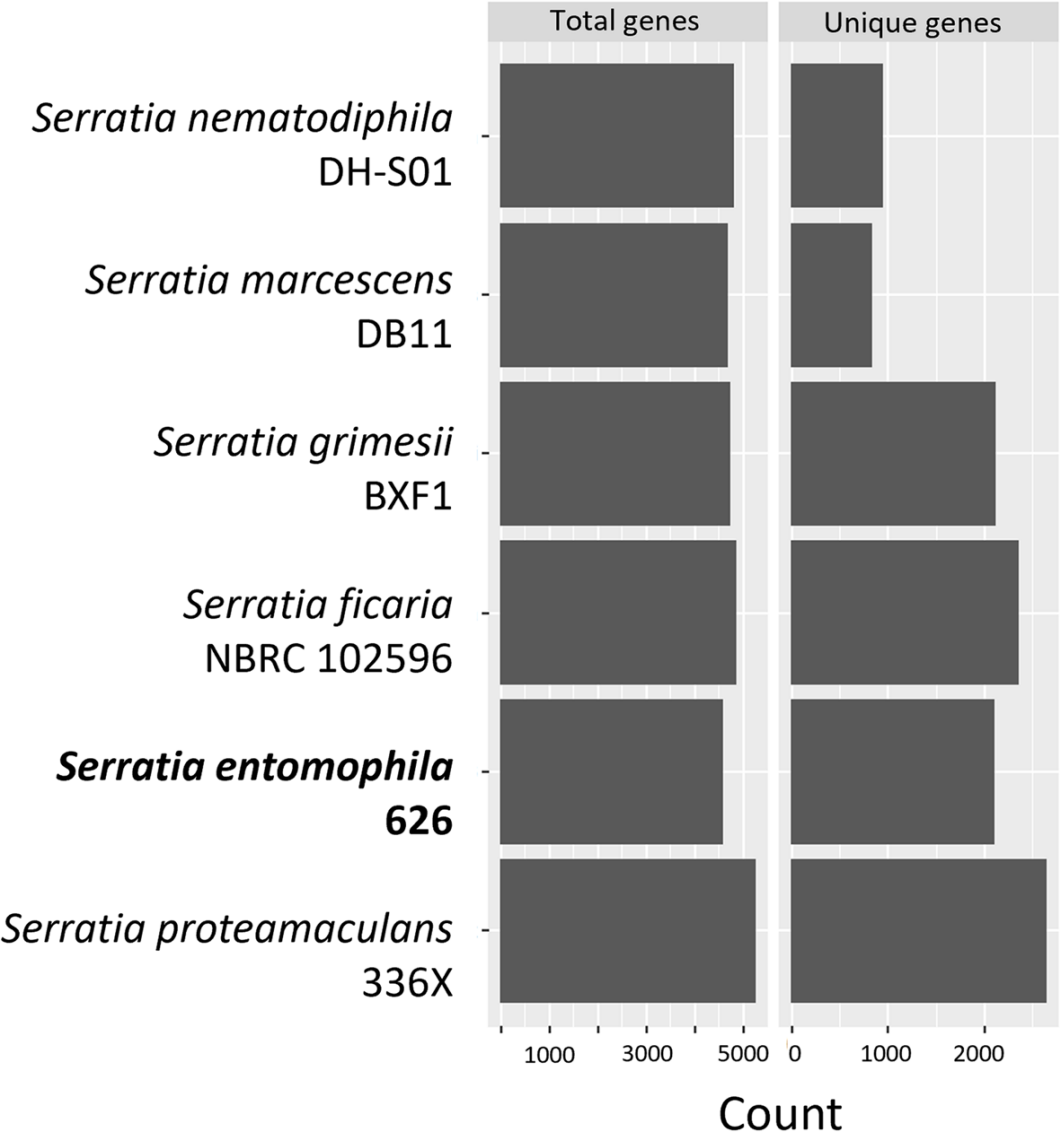
Total and unique genes for each *Serratia* isolate assessed. Minimum percentage of isolates a gene must reside to be defined as ‘core’ was set at the default of 95% amino acid similarity. *Serratia entomophila* 626 highlighted in bold

**Fig. 6:**
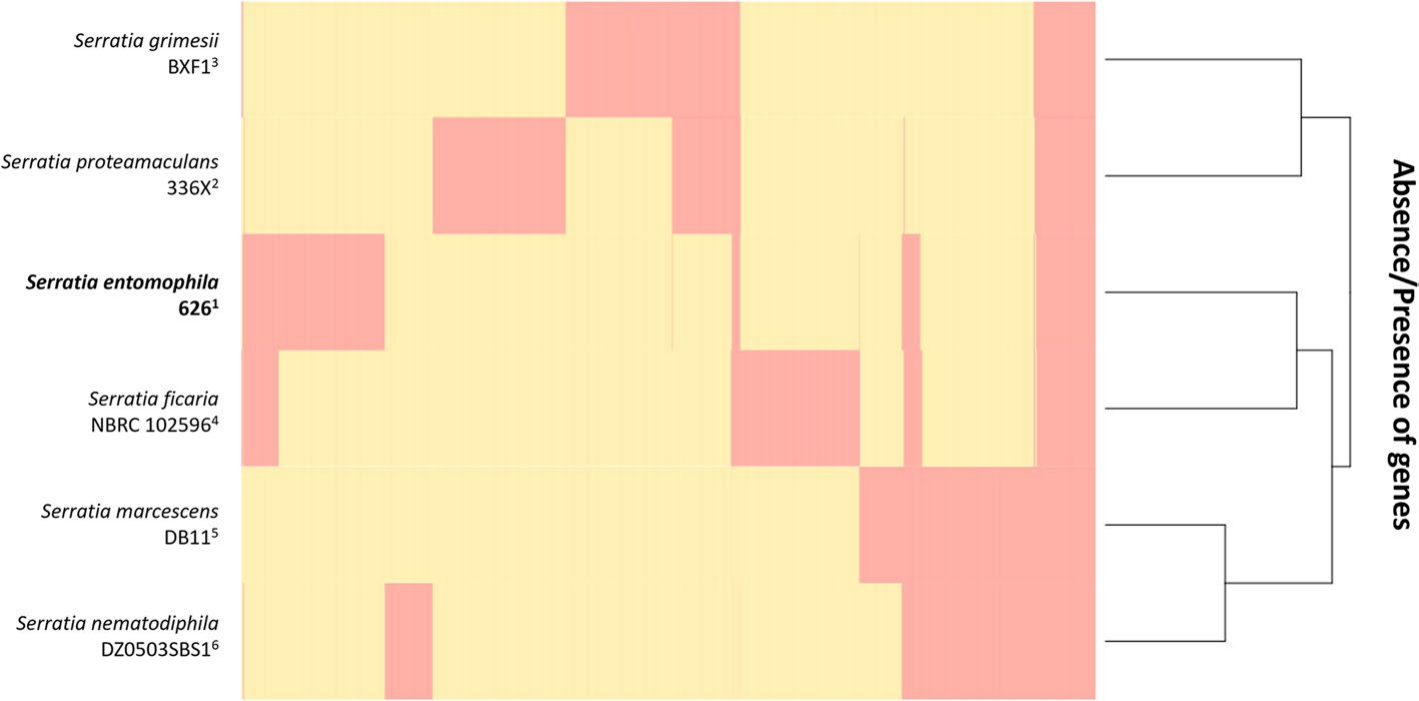
Roary alignments of *Serratia entomophila* 626 and closest related *Serratia* species. Peach denotes the presence and yellow the absence of a gene (95% cut off). *S. entomophila* 626 highlighted in bold

Determination of core genes in the *Serratia* genus was assessed by Roary through translated BlastP (95% cutoff) (Figs. 5 and 6). The average core genome (*n* = 6) was found to comprise 1,165 genes. The average gene count per isolate (*n* = 6) was 4,758 of which the average core genome comprised 1,165 genes approximating 24% of each genome. *S. nematodiphila* and *S. marcescens* encoded the fewest unique genes of the assessed *Serratia* species. Reflecting the smaller genome size, *S. entomophila* had the smallest count of total encoded genes relative to the other *Serratia* species assessed in this study (Fig. 5, Table 1). The total number of unique genes (< 95% translated amino acid similarity) identified by Roary in *S. entomophila*, *S. ficaria* and *S. grimesii* (~ 2000) was greater than in other species of *Serratia* assessed.

Roary analysis showed chromosomal similarities to *S. entomophila* were largely shared with *S. ficaria*, with a smaller region conserved across *S. entomophila*, *S. proteamaculans*, *S. grimesii,* and *S. ficaria* (Fig. 6)*.* The genomes of *S. marcescens* and *S. nematodiphila* showed greater differentiation to *S. entomophila* 626 but were largely similar to each other. Clustering phylogeny generated by Roary supports earlier 16S phylogeny/ FGD analysis (Fig. 1A and B) and ANI (Fig. 2) revealing similarities between *S. entomophila* and *S. ficaria*.

To determine the extent of the pangenome for assessed *Serratia*, analysis was undertaken to calculate the maximum number of genes within the clade. Analysis of the pangenome of the *Serratia* spp. described a Chao statistic of 11,758, and an alpha value of Heaps Law as 0.7496, defining the *Serratia* pangenome as open.

### Comparative genomic analysis

**Fig. 7:**
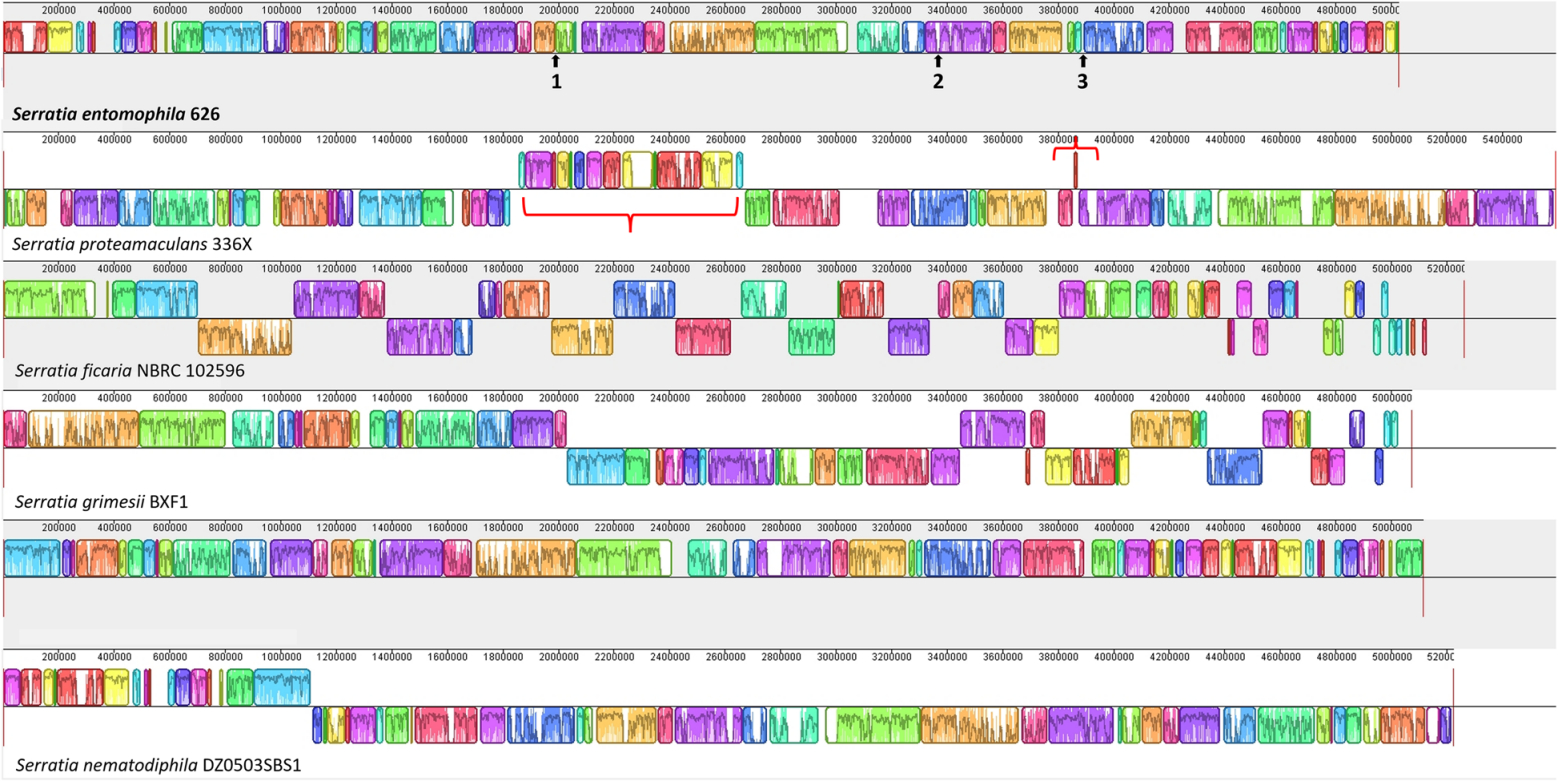
Genomic alignments of six *Serratia* spp. using MAUVE multiple genome alignment software. Blocks indicate orthologous regions- with colour maps showing the percentage nucleotide identity between each orthologous block. Blocks lying above the centre line are in the forward orientation. Blocks below the centre line are on the opposite strand and represent chromosomal rearrangements. 1) Genome location of the itaconate degradation operon in *S. entomophila* 626. 2) Location of the unique Se_DIN_. 3) Location of region encoding extracellular phospholipase A1. Parentheses denote inverted region in *S. proteamaculans* 336X relative to *S. entomophila* 626

MAUVE was used to compare large colinear blocks shared between *S. entomophila* 626 and other species of *Serratia*. Many of these clusters are putative genomic islands (listed in Table 2), or chromosomal deletions where absence of a block in an otherwise colinear section of more than two chromosomes. Large genomic rearrangements can be seen between *S. entomophila* and *S. proteamaculans* isolate 336X, with one large, inverted region in *Serratia entomophila* 626 as opposed to *S. proteamaculans* (Fig. 7). Excluding *S. ficaria,* the other assessed *Serratia* species shared large regions of uniformity over collinear blocks, with areas of low homogeneity within these areas (Fig. 7). Though sharing a large degree of orthologous gene clusters relative to *S. entomophila*, *S. ficaria* and *S. grimesii* share large collinear blocks in the opposing orientation to *S. entomophila* (Fig. 7), indicative of chromosomal rearrangements.

**Table 2:** IslandViewer4 hits predicted in *Serratia entomophila* isolate 626 with putative function assigned

Island Number^a^	Loci	Size	Predicted function^b^
1	KFQ06_00260-KFQ06_00630	51,901	Translation, transcription, carbohydrate transport and metabolism
2	KFQ06_01515-KFQ06_01775	54,030	Translation, transcription, carbohydrate transport and metabolism
3	KFQ06_02615-KFQ06_02640	4,944	Defence, transcription
4	KFQ06_02885-KFQ06_02935	8,988	Cell motility
5	KFQ06_04470-KFQ06_04485	7,213	Intracellular secretion
6	KFQ06_04445-KFQ06_04470	4,334	Intracellular secretion, transcription
7	KFQ06_05475-KFQ06_05500	4,229	Cell motility, intracellular secretion
8	KFQ06_07835-KFQ06_07890	6,651	Cell membrane biogenesis
9	KFQ06_07940-KFQ06_07970	8,424	Cell membrane biogenesis
10	KFQ06_08770-KFQ06_08805	9,015	Cell motility, defence
11	KFQ06_09240-KFQ06_09275	9,244	Transposase, translation, nucleotide metabolism
12	KFQ06_09525-KFQ06_09545	5,169	Itaconate degradation operon
13	KFQ06_11450-KFQ06_11490	8,166	General function prediction
14	KFQ06_12570-KFQ06_12605	5,633	Phage^c^
15	KFQ06_14355-KFQ06_14415	6,536	Type VI secretion system
16	KFQ06_14535-KFQ06_14565	4,071	Carbohydrate transport and metabolism; replication, recombination, and repair
17	KFQ06_14885-KFQ06_15005	32,322	Defence mechanism
18	KFQ06_15670-KFQ06_15700	4,679	Phage^c^, carbohydrate transport and metabolism
19	KFQ06_18195-KFQ06_18215	4,065	Incomplete phage^c^, transcription
20	KFQ06_18250-KFQ06_18300	7,393	Incomplete phage^c^, defence mechanisms
21	KFQ06_19095-KFQ06_19185	5,236	Defence mechanisms, cell wall biosynthesis, cell motility
22	KFQ06_20090-KFQ06_20120	16,897	Secondary metabolite biosynthesis, general function proteins
23	KFQ06_20170-KFQ06_20175	4,742	Intracellular secretion
24	KFQ06_20535-KFQ06_20760	37,287	Phage^c,^ defence, phage 2 (Fig. 3)
25	KFQ06_20865-KFQ06_20895	3,392	Phage^c^

**Fig. 8:**
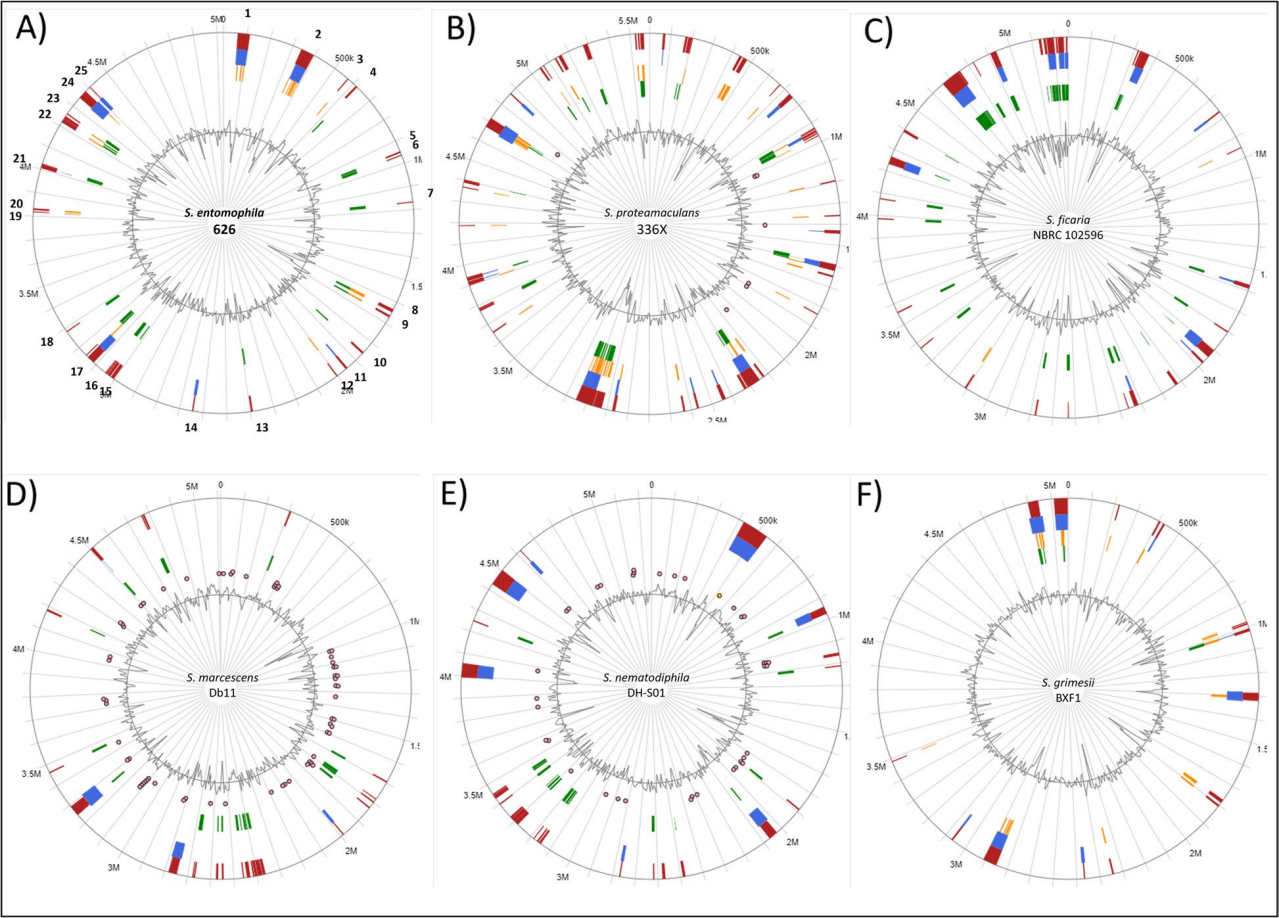
Predicted genomic island using IslandViewer4 for *Serratia entomophila* chromosome and of the genomes of the selected *Serratia* isolates. **A** Putative genomic islands for *S. entomophila* 626. Numbers correspond to genomic island with predicted COG function presented in Table 2. **B** Putative islands for *S. proteamaculans* 336X. Red indicates where a genomic island has been predicted by one of the identification tools utilised by IslandViewer (IslandPath-DIMOB, SIGI-HMM, IslandPick, Islander) where blue, orange and green represent alternate prediction tool. Pink dots show the location of homologs of antimicrobial resistance genes identified in the chromosomes of *S. proteamaculans* 336X, *S. marcescens* Db11, and *S. nematodiphila* DH-S01, where prior described island results were available in the database. The *S. entomophila* 626 itaconate degradation encoding genomic island is identified by point 12

Predicted genomic islands in *S. entomophila* 626 by any prediction method corresponding with Fig. 8

Prediction methods are Islandpath-DMOB, IslandPick, SIGI-HMM

^a^ Number corresponds to the location annotated in Fig. 6

^b^ Summary of COG categories assigned to loci

^c^ Predicted using Phaster

Assessments of genomic islands of the selected *Serratia* genomes by IslandViewer revealed *S. entomophila* 626 has 25 predicted chromosomally encoded islands (Table 2) compared to the 40 predicted islands in *S. proteamaculans* 336X and of the other assessed *Serratia* isolates (Fig. 8). These include phage remnants, toxin-antitoxin systems, anti-bacterial defensive genes, and secretion systems (Table 2). The prediction of these islands corresponds to the identification of unique regions and phages in *S. entomophila* 626 identified by BlastP against other species of the *Serratia* genus (Fig. 3). Five of the predicted islands in *S. entomophila* 626 encoded proteins with COG function attributed to defense, where one was a predicted phage. Region 3 (Fig. 8A) encoded an HRH endonuclease with no further similarity BlastP hits within the *Serratia* genus. Region 10 encoded two defense mechanism associated proteins alongside additional fimbriae proteins. One of the two defense associated proteins was a predicted hypothetical. BlastP analysis showed 84.5% identity to an addiction module antitoxin (accession: 0A240BVB2) from *S. ficaria*, whereas the second was a putative efflux pump protein. Through this analysis, the latter described 626 itaconate degradation operon (Fig. 7 labelled ‘1’; Fig. 8, Island 12), which is absent in the other assessed *Serratia* genomes, based on % G + C content IslandViewer predictions and absence within the genus is predicted as a genomic island.

Comparison of the GC skew revealed greater variability in *S. proteamaculans* 336X than in *S. entomophila* 626 (Fig. 8A and B). Based on IslandViewer, *S. entomophila* 626, *S. proteamaculans* 336X and *S. ficaria* encode a greater number of predicted genomic islands than *S. marcescens*, *S. grimesii* and *S. nematodiphila*, with *S. grimesii* encoding the least (Fig. 8).

**Fig. 9:**
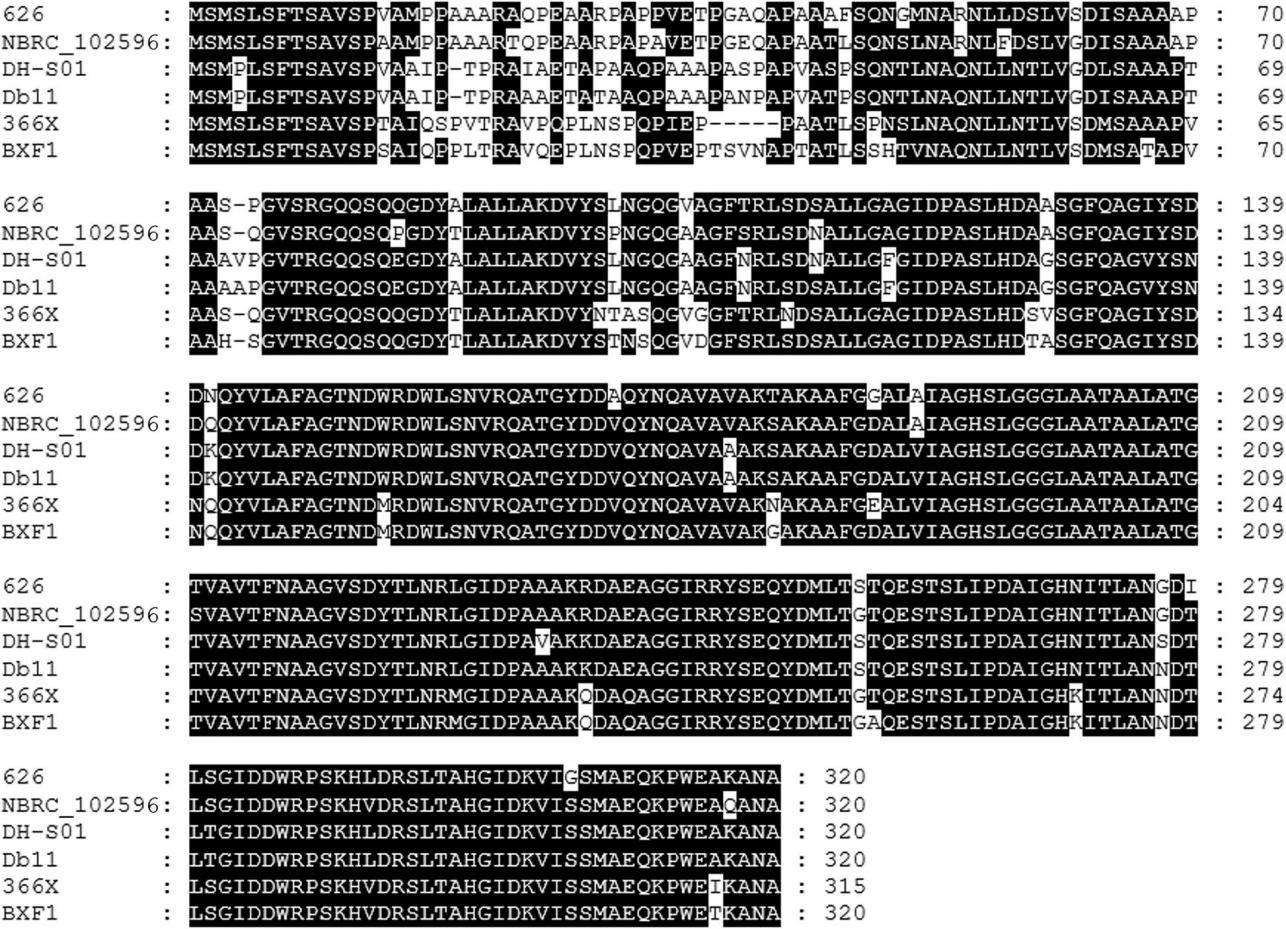
Amino acid alignment of predicted phospholipase A1 from across the *Serratia* genus. *S. entomophila* 626 (CP074347), * S. ficaria* NBRC 102596 (NZ_BCTS00000000.1), *S. nematodiphila* DH-S01 (NZ_CP038662.1), *S. marcescens* Db11 (NZ_HG326223.1), *S. proteamaculans* 336X (NZ_CP045913.1), *S. grimesii* BXF1 (LT883155). GenBank protein accessions shown in brackets

Assessments of the selected species for phage-like elements using the Phaster phage search tool revealed that the *S. entomophila* isolate 626 encoded two predicted intact (18.9 Kb and 40.4 Kb) and two incomplete phage regions (7.9 Kb and 33.3 Kb). IslandViewer predictions revealed PHAGE_Escher_500465_1_NC_049342 region in *S. entomophila* 626 comprised two smaller islands (annotated as 19 and 20 in Fig. 8) with a combined length of ~ 17 Kb. These two smaller islands encode mostly hypothetical proteins, and the Phaster predictions include adjacent fimbriae encoded genes. Across the genus, homogeneous regions span between 9.3 Kb and 14.9 Kb with minimum 67.9% pairwise DNA sequence identity to other *Serratia* isolates (Fig. 7, label 3). Nucleotide alignments of the shared region sequence identity of Escherichia phage 500465.1 ranged from 84.0% in *S. grimesii* to 92.9% in *S. ficaria*. This region encodes a DUF2974 domain-containing protein (KFQ06_18140), where Blast analysis revealed sequence homology to the extracellular phospholipase A1. Comparison of the extracellular phospholipase A1 amino acid sequences showed homology relationships to *S. entomophila* similar to that of the phylogenetic and ANI analyses, where *S. ficaria* showed the highest amino acid similarity (Fig. 9). Unique region phage 2 (Fig. 3; Table 2, Island 24) on investigation shows most in common with phage PSP3 of *Salmonella enterica.* This region however is mostly unique, where only 15 of the *S. entomophila* phage proteins shared synteny with genes from phage PSP3.

Aside from the Escherichia phage 500465.1 region, 10 predicted prophage regions were identified in *S. proteamaculans* 336X (between 12 Kb and 63.9 Kb in length), with three intact, four incomplete and three questionable phages. Both *S. grimesii* and *S. marcescens* encoded the least predicted phages, with one intact and one incomplete phage (Table 3).

**Table 3:** Annotation of Se_DIN_ and its flanking tRNA regions in *S. entomophila* 626

Loci	Name	Minimum^a^	Maximum^a^	Length	Direction
KFQ06_16105	tRNA-Thr	17,189	17,264	76	reverse
KFQ06_16100	S26 family signal peptidase	16,409	17,095	687	forward
KFQ06_16095	Antitermination protein	15,809	16,171	363	reverse
KFQ06_16090	Holin	15,072	15,353	282	reverse
KFQ06_16085	Lysozyme	14,639	15,085	447	reverse
KFQ06_16080	Hypothetical protein	14,168	14,557	390	reverse
KFQ06_16075	Hypothetical protein	13,779	14,171	393	reverse
KFQ06_16070	Phage tail protein	13,280	13,735	456	reverse
KFQ06_16065	Phage tail protein	12,832	13,197	366	reverse
KFQ06_16060	Hypothetical protein	12,593	12,814	222	reverse
KFQ06_16055	Phage tail tape measure protein	10,309	12,600	2,292	reverse
KFQ06_16050	Phage tail protein	9,971	10,309	339	reverse
KFQ06_16045	Phage minor tail protein L	9,209	9,961	753	reverse
KFQ06_16040	C40 family peptidase	8,496	9,200	705	reverse
KFQ06_16035	Hypothetical protein	8,120	8,458	339	reverse
KFQ06_16030	Tail assembly protein	7,467	8,081	615	reverse
KFQ06_16025	DUF1983 domain-containing protein	3,832	7,413	3,582	reverse
KFQ06_16020	Tail fiber domain-containing protein	2,619	3,781	1,163	reverse
KFQ06_16015	Prophage tail fiber N-terminal domain-containing protein	613	2,622	2,010	reverse
KFQ06_16010	DinI family protein	224	466	243	forward
KFQ06_16005	tRNA-Pro	1	77	77	forward

^a^ Minimum and maximum lengths calculated from the tRNA boundaries of the putative genomic island

Unique to *S. entomophila* and not predicted by IslandViewer is an 18.9 Kb region located between 3.35 Mb-3.37 Mb of the 626 chromosome. Flanked by tRNA-Pro and tRNA-Thr, this phage-like structure is devoid of DNA packing apparatus, 5’ of which is a gene encoding a DinI protein (Fig. 3 Phage_1, Table 2 Island 18, Fig. 7 ‘2’, Fig. 8, Table 3). The predicted phage-like Din Island designated Se_DIN_ (Island 18, Fig. 8) has a lower G + C content (55.3%) relative to the chromosome to *S. entomophila* 626 (59.1%).

To define potential genomic regions that may limit HGT, chromosomal searches were undertaken to locate CRISPR-Cas and restriction-modification (R-M) systems. Neither *S. proteamaculans* 336X nor *S. grimesii* DXF1 encoded R-M systems, whereas type 1 R-M systems were ubiquitous across other isolates. *S. marcescens* Db11 (Type 3 R-M) and *S. nematodiphila* DH-S01 (Type 2) were unique in the additional R-M system types present on the chromosome. Three of the six assessed *Serratia* encoded putative CRISPR-Cas systems, with a greater number in *S. grimesii* DXF1 (Table 4). CRISPR arrays in *S. ficaria* and *S. proteamaculans* 336X had short candidate arrays of 1–3 spacers that may be recent or relic arrays that may not be functional CRISPR systems, as predicted through CRISPR-CASFinder. *S. grimesii* had one array with low evidence (< 4 spacers present) and two active CRISPR-Cas systems (Table 4). *S. entomophila* 626, like *S. marcescens*, contains no CRISPR-Cas systems, but similar to the other assessed *Serratia* species does encode a type I R-M system (Table 4).

**Table 4:** Predicted phage, CRISPR-Cas systems and R-M systems within the assembled chromosome of *Serratia entomophila* 626 and the selected *Serratia* spp

	Phage predictions				
Isolate	Intact	Incomplete^a^	Questionable^a^	CRISPR	R-M systems^b^
626^d^	2	2	0	0	I
336X^e^	3	4	3	1^c^	-
DH-S01^f^	1	4	0	0	I, II
DXF1^g^	1	1	0	3	-
Db11^h^	1	1	0	0	I, III
NBRC 102596^i^	0	2	0	1^c^	I

^a^ Incomplete phage lack an integrase gene, whereas questionable assigned phages do not have sufficient genes to be considered complete or functional

^b^ I = Type I R-M system, II = Type II R-M system, III = Type III R-M system,— = no R-M system

^c^ Short candidate array of one to three spacers that may not be a CRISPR array

^d^ *S. entomophila*, ^e^ *S. proteamaculans*, ^f^ *S. nematodiphila*, ^g^ *S. grimesii*, ^h^ *S. marcescens*, ^i^ *S. ficaria*

In agreement with the ability of *S. entomophila* to produce DNase, *S. entomophila* isolate 626 encoded a single non-specific endonuclease, the translated products of which have high amino acid identity with *nucA* orthologues from *S. marcescens* and *S. ficaria* (Table 5). A second endonuclease, *endA* was identified, the translated product of which shares 95.2% amino acid identity to EndA in *S. ficaria* and shares 75.3% amino acid identity with *Dickeya dadantii* NucM (Table 5).

**Table 5:** Identification of secreted nuclease from *S. entomophila* isolate 626, and its closest % similarity from BlastP

Locus	Protein/ Amino acid size	Predicted function	% identity/% similarity/% coverage	Accession
KFQ06_08805	DNA/RNA non-specific endonuclease (266)	Endonuclease	92.83/97/100 *S. plymuthica*	WP_004942831.1
KFQ06_19955	EndA (232)	Endonuclease	95.2/96/99 *S. ficaria*	WP_061798583.1

### Chromosomally encoded hydrolases and metabolites

To help define the plasmid-independent virulence of *S. entomophila* relative to the other assessed *Serratia* spp. the chromosomes were independently interrogated by using hmmsearch searching for chitinase and lipase motifs. The 626 chromosome was found to encode single copies of four chitin and one chitin-binding protein (Table 6). Most lipases identified from Pfam HMM motif searches were identified in isolate 626, which shared high amino acid similarity to the *S. ficaria* lipases (A0A240AUF3). Isolate 626 also encodes an extracellular phospholipase (KFQ06_18140) sharing 90% amino acid identity to the *S. liquefaciens* extracellular lipase A1 (A0A240CAJ3) and co-located to the Escherichia phage 500465.1 found across the genus. Other Lipases were consistently present across the genus (Table 6).

**Table 6:** Presence or absence of lipase, chitin binding and chitinases encoding genes of the assessed *Serratia* spp

Loci^*^	Protein	**626**^**1**^	336X^2^	BXF1^3^	NBRC 102596^4^	Db11^5^	DH-S01^6^
KFQ06_05725	Thioesterase I	** + **	+	+	+	+	+
KFQ06_00845	Lysophospholipase L2	** + **	+	+	+	+	+
KFQ06_00825	Phospholipase A	** + **	+	+	+	+	+
KFQ06_20210	Phospholipase C	** + **	+	+	+	+	+
KFQ06_18140	Phospholipase A1	** + **	+	+	+	+	+
KFQ06_05495	Chitin binding protein	** + **	+	-	-	-	-
KFQ06_17240	Chitinase B	** + **	+	+	+	+	+
KFQ06_00590	Chitinase A	** + **	+	+	+	+	+
KFQ06_13130	Chitinase A1	** + **	+	+	-	+	+
KFQ06_06080	Chitinase D	** + **	+	-	-	-	-

^*^ Loci number corresponds to respective protein on the chromosome of *S. entomophila*

^1^ *Serratia entomophila* 626, ^2^ *S. proteamaculans*, ^3^ *S. grimesii*, ^4^ *S. ficaria*, ^5^ *S. marcescens*, ^6^ *S. nematodiphila*

Presence or absence based on motif hits using hmmsearch

Of relevance to potential entomopathogenic properties, four chitinases (Chitinase A, A1, B and an orthologue of ChiD) were present in *S. entomophila* 626, each harbouring a glycoside hydrolase family 18 domain (Table 7). Chitin-binding protein GbpA was only found in *S. entomophila* 626 and *S. proteamaculans* 336X. Excluding chitinase A1, which was absent in *S. ficaria* and ChiD*,* two additional encoded chitinases were present across the assessed *Serratia*. The loci KFQ06_20145 encoding a protein with a hydrolase family 18 domain shares low amino acid similarity with a predicted lipoprotein (Table 7).

**Table 7:** Chitin-associated genes and their respective functions determined through UniProt

Locus	Protein name	Predicted function	Size (AA) *S. entomophila*	% identity/Similarity	Accession
KFQ06_00590	ChiA	Chitinase A	564	99.11/100 *S. plymuthica*	WP_135314641.1
KFQ06_17240	ChiB	Chitinase B	500	95.39/100 *S. plymuthica*	WP_126484406.1
KFQ06_13130	ChiA1	Chitinase A1	427	91.78/100 *S. plymuthica*	WP_212560081.1
KFQ06_06080	Chitinase	Chitinase D	481	90.31/100 *S. plymuthica*	WP_126480889.1
KFQ06_20145^a^	Glycoside hydrolase family 10 protein	Putative lipoprotein	427	67.61/100 *Yersinia pseudotuberculosis*	WP_050092925.1

^a^ Glycoside family 18 domain identified in hmmsearch marked as speculative

Analysis of potential secondary metabolites produced by *S. entomophila* 626 via antiSMASH revealed seven predicted secondary metabolite clusters (Table 8). Each was able to be assigned a candidate gene cluster type and only one (cluster 7) matched with high similarity to a known cluster, with a cluster hit of 77% (aerobactin). Three non-ribosomal peptide synthase (NRPS) regions were detected with one at 3.8 Kb sharing 30% cluster similarity to turnerbactin synthases. The remaining three clusters identified were identified as a putative hserlactone cluster, betalactone, and a thiopeptide synthase (Table 8).

**Table 8:** Summary of AntiSMASH analysis for predicted secondary metabolite cluster in *Serratia entomophila* 626

Loci	Type	Size (bp)	Similarity (AntiSMASH ClusterBlast)
KFQ06_00085-KFQ06_00160	Hserlactone	20,675	100% *Serratia proteamaculans* B-41162 NRRL^a^
KFQ06_01315-KFQ06_01565	NRPS	70,719	30% turnerbactin, 66% *Serratia ficaria* NCTC12148
KFQ06_03670-KFQ06_03765	Betalactone	25,659	95% *Serratia rubidaea* 1122^a^
KFQ06_04535-KFQ06_04585	Siderophore	14,438	77% aerobactin *Xenorhabdus,* 26% *Serratia ficaria* NCTC12148
KFQ06_08385-KFQ06_08465	Thiopeptide	26,454	14% O antigen, 100% *Serratia ficaria* NCTC12148
KFQ06_11775-KFQ06_11840	Redox cofactor	22,163	13% lankacidin C, 15% *Pluralibacter gergoviae* FDAARGOS 186
KFQ06_14760-KFQ06_15050	NRPS	62,597	5% ravidomycin, 84% *Serratia marcescens* 4928STDY7387938
KFQ06_20030-KFQ06_20175	NRPS T1PKS	57,637	35% *Serratia ficaria* NCTC12148^a^

^a^ Results displayed from AntiSMASH show identification of similar cluster in other species, where percentage shows the percentage of genes showing nucleotide similarity

### *Serratia entomophila* encoded itaconate degradation cluster

**Fig. 10:**
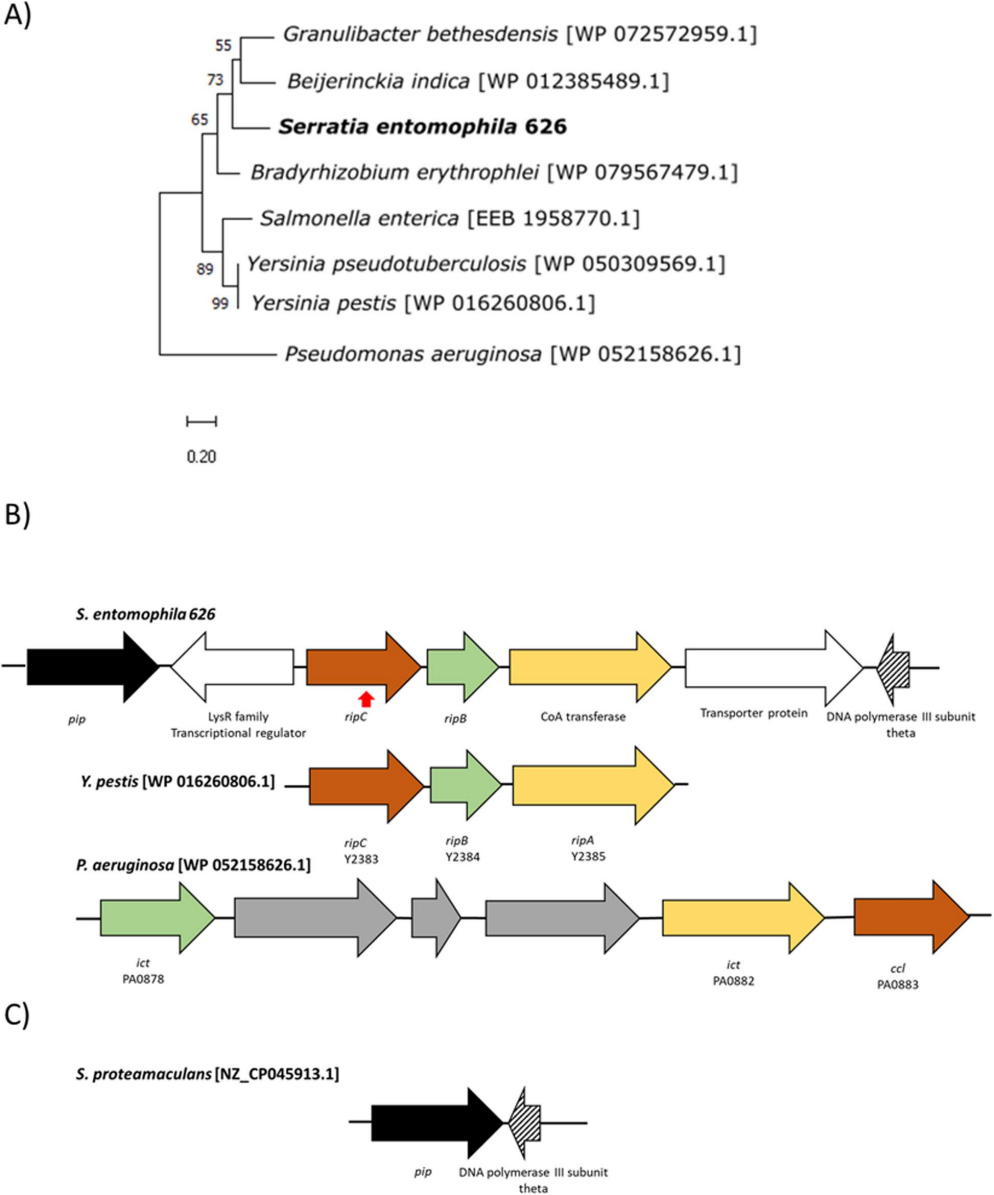
Gene synteny of the itaconate degradation pathway operon. **A** Maximum likelihood tree of RipC amino acid sequence from *Serratia entomophila* (bold) alongside seven other gene homologues found through BlastP. Scale bar represents 20% genetic variation. Bootstrap values above 50% are shown. **B** *ripABC* synteny and gene arrangement with the depicted itaconate operons- the three gene *Yersinia pestis* and six gene *Pseudomonas aeruginosa* operons. Colours indicate genes with the same functional prediction as *S. entomophila*, refer Table 8 for annotations. Red arrow under *ripC* denotes the mutated gene. **C** *S. proteamaculans* co-location of *pip* and DNA polymerase III subunit theta, where in S*. entomophila* 626 the itaconate degradation region is positioned. GenBank protein accessions shown in brackets

Based on the ability of *S. entomophila* to utilize itaconate as a sole carbon source and the predicted itaconate degradation operon residing as a predicted genomic island 12 (Fig. 3 unique region 6; Fig. 4, Fig. 8) absent from the other assessed *Serratia* species, the *S. entomophila* itaconate operon was assessed for its potential role in virulence. As listed in Table 9, the itaconate region comprises four genes: i) coenzyme A (CoA) transferase, ii) *ripC* encoding l-malyl-CoA lyase, iii) *ripB* the translated product of which encodes a mesaconyl-CoA hydratase, and iv), a putative transporter protein. Based on gene synteny the *S. entomophila* itaconate degradation region is most like the *Y. pestis ripABC* operon identified as *ripC, ripB,* and CoA transferase (Fig. 10B) and shares varying levels of amino acid similarity to the translated components of the *Y. pestis rip* operon (Table 9). RipC is more diverged, sharing only 57% amino acid similarity with the *Y. pestis* RipC ortholog. RipC phylogeny of BlastP closest relative proteins showed that the *S. entomophila* 626 RipC protein is closely related to that from the nitrogen-fixing soil bacterium *Beikerinckia indica* (Fig. 10A). Phyre.2 analysis of the translated product of the KFQ06_09545 located 3’ of *ripC* revealed 90% structural identity to a family member of the NADC transporter protein (Table 9). Located in the opposing orientation 3’ of the *S. entomophila* itaconate operon is a predicted LysR regulator (KFQ06_09525). Five prime (DNA polymerase III subunit theta, KFQ06_09550) and 3’ (*pip,* KFQ06_09520) genes flanking the *S. entomophila* itaconate region are co-located in the non-itaconate encoding *S. proteamaculans* 336X genome (Fig. 10C). No IS or repeat elements were detected at the periphery of either the *S. entomophila* 626 or the *Y. pestis ripABC* predicted itaconate operons. Relative to 626 itaconate encoding region with a % G + C of 59.1%, the *Y. pestis* KIM10 + itaconate clusters % G + C was lower (51.2%), supporting the hypothesis of its acquisition by HGT.

**Table 9:** Results of the BlastP analysis of the *Serratia entomophila* and *Yersinia pestis* itaconate degradation encoding operon, showing closest related ortholog and origin species

Loci	ORF	A.A length	% identity/ % similarity /coverage, protein domain	Function	Organism
KFQ06_09525	LysR family regulator	299	66.7/64.7/97	Transcriptional regulator	*Rhizobium leucaenae* WP_184804500.1
KFQ06_09530	RipC	277	63.3/60.7/96	Itaconate degradation C–C-lyase	*Bradyrhizobium erythrophlei* WP_079567479.1
Y2383^b^	RipC	280	100/100/100	Itaconate degradation C–C lyase	*Yersinia pseudotuberculosis* WP_002212068.1
KFQ06_09535	RipB	175	85.7/85.7/100	(R)-specific enoyl-CoA hydratase RipB/Ich	*Bradyrhizobium eklanii* WP_209944478.1
Y2384^b^	RipB	216	99.4/82.5/83	MaoC family dehydratase	*Yesinia pseudotuberculosis* MBO1554006.1
KFQ06_09540	CoA transferase	393	74.8/72.5/98	CoA transferase	Unclassified *Pseudomonas* WP_008146597.1
Y2385^b^	RipA	440	100/100/100	Itaconate CoA- transferase	*Yersinia pseudotuberculosis* WP_161597823.1
KFQ06_09545	Hypothetical	430	55.5/55.5/97	Transporter protein^a^	*Pasteurellaceae* bacterium TNH05083.1

^a^ Prediction of Phrye

^b^ *Yersinia pestis* genes from isolate KIM10

### Characterization of the *S. entomophila* itaconate region

**Fig. 11:**
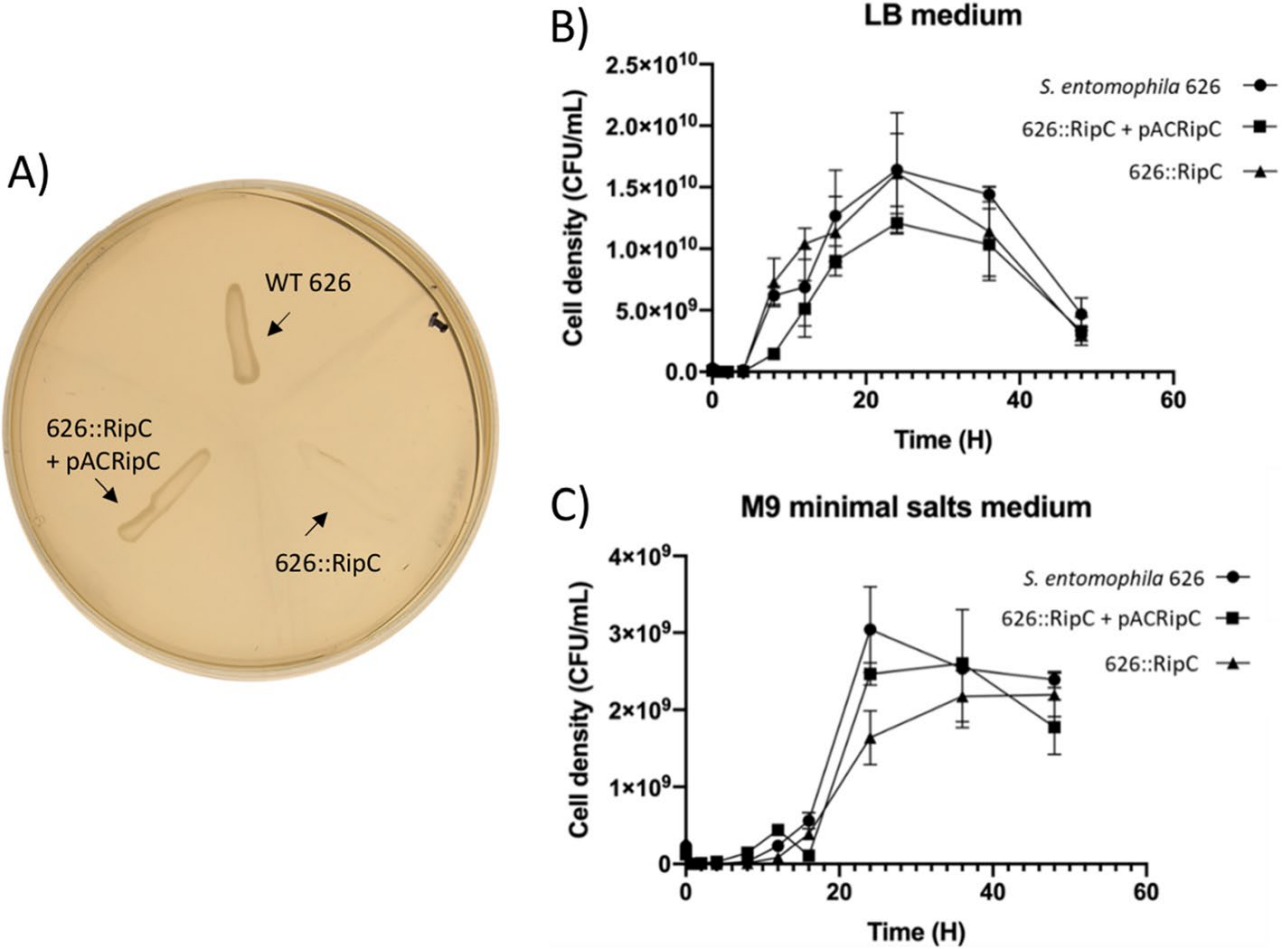
Growth of WT 626, 626::RipC and complemented *ripC* gene in optimal and stress conditions. 48 h growth curves in triplicate with standard error shown. A) growth of wildtype 626, the 626::RipC mutant and its trans-complemented derivative 626::RipC pACRipC on itaconate agar.B) in LB broth and C) M9 minimal (glucose) broth

Based on the role of the itaconate degradation operon in the utilisation of itaconate as a carbon source [28], it is plausible that this region may be advantageous to *S. entomophila* in a specific niche. As expected, the 626::RipC mutant was unable to grow on ITA agar plates (Fig. 11A) validating the role of the *ripC* gene in itaconate utilization. This loss of ITA utilization was able to be restored through the *trans* complementation by pACRipC with 626::RipC, where similar growth to that of the 626 strain was observed on ITA agar media (Fig. 11A).

To determine any metabolic benefit the itaconate degradation operon may confer to the growth of *S. entomophila*, the growth kinetics of *S. entomophila* 626 and 626::RipC were independently assessed over a 48 h duration in LB and then M9 (glucose) broth. Assessments of the resultant growth curves found no difference in the growth of *S. entomophila* 626 and 626::RipC in LB broth (Fig. 11B). In M9 minimal broth (glucose), the rate of growth of 626::RipC was reduced in the lag and exponential growth phase. The CFUs of 626::RipC and 626::RipC + pACRipC plateaued by the 48 h time points with 2.19 × 10^9^ and 1.98 × 10^9^ CFU/ mL respectively, where wildtype *S. entomophila* 626 achieved higher cell numbers of 2.39 × 10^9^ CFU/ mL (Fig. 11).

### Larval co-infection assays

To determine if 626::RipC may impair the infectivity to challenged *C. giveni* larvae, the lethal concentration (LC_50_) and lethal time (LT_50_) of disease were determined for 626::RipC and *S. entomophila* 626 (Table 10). Initial bioassay assessments of *C. giveni* larvae separately challenged with isolates 626 or 626::RipC defined LC_50_ of 626 to be approximately tenfold lower than that observed with 626::RipC (Table 10). Assessment of the LT_50_ where larvae were dosed with ~ 7 × 10^7^ CFU revealed an LT_50_ of 3 days in WT *S. entomophila* 626 and 4 days in the 626::RipC mutant.

**Table 10:** Bioassay LC_50_ and LT_50_ data with standard error on the mean for mutant 626::RipC and *Serratia entomophila* 626 controls. Results were determined from day 12 observations. *P* values (Fisher’s exact), with statistical significance to the negative control, are highlighted in bold

Isolate	LC_50_^a^ (± standard error)		LT_50_^b^ (Days)	Diseased (%) ± standard error		Mortality (%) ± standard error		Combined (%) ± standard error	
626	1.95 × 10^5^	± 9.8 × 10^5^	3	70.83	± 9.47 **(< 0.001)**	29.16	± 9.47 (0.492)	100	± 0.0 **(< 0.001)**
626::RipC	2.01 × 10^6^	± 1.4 × 10^6^	4	75	± 9.02 **(< 0.001)**	25	± 9.02 (0.742)	100	± 0.0 **(< 0.001)**

^a^ Undertaken via Probit analysis

^b^ Statistical Survival analysis

**Fig. 12:**
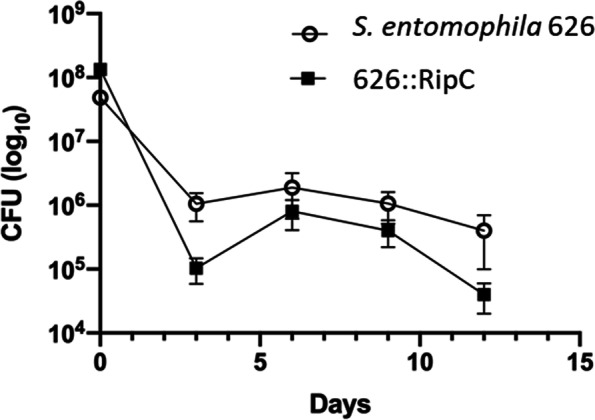
In vivo competitive growth experiment. In vivo growth curve of a 12 day of 50:50 inoculants of WT 626 and its itaconate mutant derivative 626::RipC in challenged *C. giveni* larvae, represented on a log_10_ scale. CFU log_10_ results for each isolate recorded in triplicate for three-day intervals

To determine any in vivo potential competitive advantage of *S. entomophila* 626 over 626::RipC a co-infection assay of *C. giveni* larvae with both strains was carried out and relative cell numbers 12 days post-challenge assessed (Fig. 12). Although inoculation CFU for WT 626 (6.2 × 10^9^ /mL) was slightly lower than for the itaconate mutant (9.6 × 10^9^ /mL), CFU of *S. entomophila* 626 re-isolated from in vivo macerate samples remained (~ 1 × 10^6^ CFU) higher than for the mutant strain. At 3 days post-challenge, the cell numbers for both strains significantly differed (*P* = 0.013), where WT 626 showed an advantage in the establishment of the larvae post-challenge over the mutant (Fig. 12). Unlike the 626::RipC mutant, WT 626 remained at a relatively stable cell density (~ 10^6^ CFU per larvae) over the duration of the 12-day competition assay. From day 6 until day 12, the 626::RipC cell number declined by 95%, dropping to ~ 5 × 10^4^ CFU per larvae by day 12. While the endpoint difference did not significantly differ (*P* = 0.057), WT 626 (10^4^ CFU per larvae) was trending towards a growth advantage against the 626::RipC mutant with a log fold difference in cell numbers between days 3 to 12 of the bioassay*.*

## Discussion

The complete genome of the *S. entomophila* BioShield® isolate 626, used for the control of *C. giveni* larvae in New Zealand, was sequenced and described in this study.

As determined through Roary, the core genome of *Serratia* genomes assessed in this study (*n* = 1165) is 24% of the overall chromosome size. The inclusion of additional and more varied *Serratia* spp. would likely decrease the core genome of *Serratia* spp., as suggested by the value of alpha in Heap’s law describing an open pangenome. Chao’s statistic [29] however, defines the upper bounds of the pangenome as 11,758 genes. Close relationship predictions of the assessed *Serratia* by ANI (> 62%) are within the genus boundary suggested by Kim et al. [30] and a large core genome suggests that adaptive evolution to host and environment in the *Serratia* genus is mediated by acquisition of DNA through HGT and chromosomal rearrangements within the genus. Of these relationships, *S. entomophila* shares the highest ANI with *S. ficaria* (91.1%). This was further supported by 16S and core genome phylogenies, showing *S. ficaria* is the closest related *Serratia* species identified to *S. entomophila* 626. COG assessments of the number of encoded phage-associated genes revealed *S. entomophila* (*n* = 69) and *S. nematodiphila* (*n* = 71) are second to *S. proteamaculans* (*n* = 132). *S. grimesii, S. marcescens* and *S. ficaria* encoded 34, 49 and 32 respectively. This correlates with the predicted number of phages within the genus, where *S. proteamaculans* had the most phage elements and *S. marcescens, S. ficaria* and *S. grimesii* the fewest. Of interest is the predicted *S. entomophila* island which has remnant orthologues with high nucleotide identity in the other assessed *Serratia* genomes. The identification of a phospholipase A1 (KFQ06_18140) associated with this region high nucleotide identity across the assessed *Serratia* species alludes that earlier DNA acquisition facilitated pathogenic development within the *Serratia*. Quantification of chromosomally encoded accessory enzymes with a role in virulence in *S. entomophila* 626 revealed lipases were uniform across the genus. Amino acid sequence comparison shows 84% pairwise identity of phospholipase A1 across all six isolates, with highest amino acid identity (90%) between *S. entomophila* 626 and *S. ficaria.*

Although comparable in number to *S. ficaria* NBRC 102596 the *S. entomophila* 626 chromosome encodes more predicted genomic islands than *S. grimesii* DXF1 and *S. marcescens* Db11 and less than *S. proteamaculans* 336X and *S. nematodiphila* DH-S01. This suggests *S. entomophila* has reduced genomic plasticity compared to *S. proteamaculans* 336X, but not relative to the other examined species. The increased number of predicted *S. entomophila* genomic islands relative to some members of the genus may mean that the *S. entomophila* genome diversified before its association with *C. giveni* larvae. Evidence for this may be the presence of the species unique Island Se_DIN_ which encodes a predicted phage but was devoid of DNA packaging or capsid genes and encodes a DinI protein. In *E. coli* DinI physically interacts with RecA to shut off the initiation of the SOS response. Of note the *S. entomophila* Afp is regulated by the *rpoS* SOS response regulator [31], therefore the expression of the Se_DIN_ associated DinI may affect gene regulation in this bacterium including that of the Afp.

Assessment of R-M systems located on the chromosomes showed the prevalence of type I systems within the genus. CRISPR-Cas systems [32], in addition to R-M systems [33], are thought to be inhibitors of the flow of HGT in bacteria. CRISPR-Cas systems have been shown to inhibit conjugation transformation and phage integration. Previous research [32] found a *Bacillus cereus* CRISPR-Cas actively impeded HGT, with active systems correlating with fewer mobile genetic elements. New evidence suggests that phage transduction is promoted by CRISPR-Cas adaptive immune systems in phage-resistant and sensitive populations of *Pectobacterium atrosepticum* by limiting wild-type phage replication and promoting transduction in phage-sensitive and resistant populations [34]. This suggests that the role of CRISPR-Cas systems is more complex than simply inhibiting HGT, and any effect the presence of CRISPR arrays has on HGT cannot be fully defined via in silico analysis.

Using IslandViewer, fewer predicted genomic islands were identified in those *Serratia* chromosomes characterised by the presence of one or two R-M types or a CRISPR-Cas system. For example, *S. grimesii* BXF1 encodes two predicted chromosomal CRISPR-Cas systems with a low prediction score to a third CRISPR-Cas array and encodes the fewest putative genomic islands. Active CRISPR-Cas systems have been implicated as a potential constraint to HGT as demonstrated in *Pseudomonas aeruginosa*, where the presence of an active CRISPR-Cas system correlated with the reduced number of putative genomic islands [35]. No intact CRISPR-Cas were identified in *S. entomophila* 626, *S. proteamaculans* 336X or *S. marcescens* Db11.

In relation to the potential speciation of 626, a single Type 1 R-M system was identified on the chromosome of *S. entomophila* 626 which, through its ability to cleave foreign DNA, could limit acquiring of foreign DNA. Further to this the production of extracellular DNase by *S. entomophila* [16] will likely reduce the opportunity for cell surface HGT DNA acquisition [36].

*S. entomophila* is the only species within the *Serratia* genus to encode an itaconate degradation operon, where dissimilar % G + C content and putative genomic island location prediction alludes to species-specific acquisition. The 626::RipC mutant showed a slight growth lag in challenged larvae but did not affect the disease development. However, *C. giveni* larvae challenged with 626 and 626::RipC revealed that 626 had an initial competitive advantage, as the predominant strain isolated from larvae. Though the 626::RipC mutation did not affect the virulence capacity of the bacterium to challenged *C. giveni* larvae, the increased fitness of 626 over 626::RipC observed through co-infection and noted in M9 minimal broth, suggested that a substrate of gene products of the itaconate operon is present in *C. giveni.* In an ecological context, it is also plausible that the 626 encoded pathway may utilize fungal-secreted itaconate as a carbon source [37]. In this context a synergistic relationships between *S. entomophila* and entomopathogenic fungi were previously reported by Glare [38], and saprophytic fungi are often associated with the cadavers of amber disease-affected larvae. The potential utilization of fungal derived itaconate by *S. entomophila* through post amber disease saphrophytic decay would prolong the bacterium’s survival external to the host and therefore warrants further investigation.

## Conclusions

The complete chromosomal sequence of *S. entomophila* isolate 626 will enable future analysis exploring the relationship of *S. entomophila* with *C. giveni* larvae. Relative to other grass grub and manuka beetle active pathogens such as *S. proteamaculans* AGR96X and *Yersinia entomophaga* which cause mortality within 3–10 days post-challenge [39], *S. entomophila* is a more benign pathogen, with infection taking 3–4 months before mortality [8]. The chronic nature of *S. entomophila* mediated amber disease would enable *S. entomophila* to exist in a non-competitive niche. By reducing its DNA acquisition potential through fewer microbial associations, *S. entomophila* could then decreased production burden of a highly active pathogen [40]. Direct support for this was provided by Dodd [13] and Claus et al. [14] who demonstrated the genome of entomopathogenic *S. proteamaculans* is more heterogeneous than for *S. entomophila*.

The presence of R-M systems and fewer genomic islands combined with previous assessments of Dodd et al. [41] and Jackson et al. [8] support the hypothesis that the *S. entomophila* genome may reflect a lifestyle adaptation suiting an association with *C. giveni* larvae. Further exploration of isolates of *S. entomophila* would facilitate determining the evolutionary relationship with *C. giveni* and allude to whether genome reduction is underway*.*

## Methods

### Culture and genome sequencing

Cultures were grown in 3 mL of Luria–Bertani (LB) broth for 16 h at 37 °C for *Escherichia coli,* and 30 °C for *S. entomophila* 626 and its derivatives, in a Ratek orbital incubator at 250 rpm. Antibiotic concentrations used for selection and counterselection of *S. entomophila* 626 and its derivatives were tetracycline 30 μg mL^−1^ kanamycin 100 μg mL^−1^, chloramphenicol 90 μg mL^−1^ and for *E. coli* were ampicillin 100 μg mL^−1^, chloramphenicol 30 μg mL^−1^, kanamycin 50 μg mL^−1^, and tetracycline 30 μg mL^−1^.

Luria Bertani and M9 (glucose) minimal growth media were prepared as described in Elbing et al. [42]. To validate the presence of *S. entomophila,* selective caprylate-thallous agar (CTA), DNase, and itaconate (ITA) plates were prepared and used as outlined by O'Callaghan [16]*.*

For standard growth curves, cultures were initially grown for approximately 16 h (~ 1 × 10^9^ CFU). Starting concentrations were then equalised through the addition of LB broth to a CFU of ~ 1 × 10^7^ CFU/mL and the CFUs validated by serial dilution. Five hundred µL of the equilibrated culture was then pelleted and resuspended in 500 µL phosphate-buffered saline (PBS) before independently inoculated into three flasks containing either 50 mL of LB broth or M9 (glucose) per isolate. CFUs were determined using serial dilutions prepared in PBS buffer by taking 1 mL samples at the time of inoculation and 1, 2, 4, 8, 16, 24, 26, and 48-h post-inoculation (hpi). OD_600_ was measured in triplicate at each of these time points using a Bio-Rad SmartSpec Plus Spectrophotometer.

### DNA preparation and sequencing

Standard molecular techniques were undertaken as outlined by [43]. Genomic DNA extractions were performed with the Bioline ISOLATE II Genomic DNA kit (Meridian Bioscience, UK) following the manufacturer’s instructions. For amplification of genetic regions, Roche platinum *taq* DNA polymerase was used according to manufacturer’s instructions. Vectors, primers, and amplicons used in this study are listed in Table 11. Plasmid vector DNA and PCR amplicons were purified using the respective Roche high pure plasmid isolation kit or the Roche high pure PCR product purification kits (Roche Diagnostics GmbH, Mannheim, Germany). Yield and purity were determined using agarose gel electrophoresis and NanoDrop 2000 Spectrophotometer (Thermo Scientific).

**Table 11:** Primers used in this study

Primer	Sequence (5’-3’)^a^	Amplicon size (bp)	Amplicon
ItaF ItaR	aaatctagaGGTTTGTACCCGCCGTTAGCAG aaatctagaCTCGCCCTTGACGGCCTGATCG	3677	Ita-RipC
tetNcoI_f tetNcoI_r	aaaccatggGAGTTAGTCTTGAAGTCATGCGC aaaccatggGCATTCACAGTTCTCCGCAAG	1611	Tetracycline cassette
rip_f rip_r	gatatcCAGATCATCGAATCCCACCGT gatatcGTGGTTGGCGCATCTCCC	1443	*ripC*
M13F M13R	GTAAAACGACGGCCAGT GCGGATAACAATTTCACACAGG		

^a^ Lower case denotes the addition of a poly-A tail where restriction enzyme sites are underlined

Genomic DNA was sequenced at Macrogen Korea (South Korea) using the PacBio RSII system with 10 Kb SMRTbell library kit. Illumina DNA sequencing was performed by Macrogen Sequencing Service. PacBio RSII sequencing generated 104,172 reads with an average read length of 12 Kb. Sequencing coverage for isolate 626 was ~ 80X.

PacBio FASTQ reads were assembled using Canu [44] to formulate complete genomic contigs before being corrected using Pilon against *S. entomophila* Illumina sequences [44, 45]. The assembled contigs were trimmed using Circlator to remove overhangs in circular DNA assemblies [46]. The resultant plasmid assembly [15] (Accession: NC_002523) was identified by size, and BlastN [47] for similarity to *S. entomophila* plasmid pADAP, and removed from the genome assembly. CheckM was used to assess the quality of the microbial genome [48], with an estimated genome completeness of 99.88% and no contamination identified in the sequence of *S. entomophila* 626. The *S. entomophila* 626 chromosome is deposited in GenBank with accession CP074347. Reference sequences from GenBank of the five reference *Serratia* genomes used in the study are listed in Table 2. Genome annotation was performed by PROKKA Rapid Prokaryotic Genome Annotation software and through GAMOLA2 [49, 50]. COG assessments of *S. entomophila* isolate 626 were compared to five reference *Serratia* type strains (Table 2) using the latest COG database [51]. The results were then used to construct a genome atlas for *S. entomophila* isolate 626 using Genewiz [52], utilizing BlastP with a custom Blast database comprising the five reference strains, COG annotations and in-house software.

### Comparative genomics

Core genome analysis was undertaken using ROARY pangenome pipeline, where nucleotide sequences provided from.gff3 annotations were converted into amino acid sequences and undergo all vs all BlastP. Protein percentage sequence identity were set at default cutoff values of 95% [53]. Pangenome analysis was undertaken using the R package micropan [54]. Large-scale genomic changes of locally collinear blocks were assessed using MAUVE [55]. Hmmer3 [56] hmmsearch function was used in parallel with the Pfam motif database to search the genome of *S. entomophila* 626 for lipases, chitinases, and DNases. Default parameters were used for cutoff threshold (E-value = 10.0) to display all potential hits. Genomic islands were predicted using IslandViewer4 [57]. The PHASTER server (https://phaster.ca/) was used for detection of chromosomally encoded phage regions [58]. RAST annotations [59] were searched for restriction-modification (R-M) systems, and CRISPR regions were identified using CRISPRfinder [60].

### Phylogenetic analysis

16S rDNA sequences from selected type isolates of *Serratia* spp. were extracted from GenBank (refer to Table 2 for selected *Serratia* spp. and accession numbers). 16S rDNA genes were aligned using ClustalW and plotted using maximum likelihood phylogenetic inference in MEGA7 software [61].

Core genome phylogeny was inferred using whole genomes of 13 representative strains of *Serratia* available on the Genbank repository, included in this analysis was the genome of recently deposited *S. entomophila* A1 type strain. To identify single-copy orthologous groups, Orthofinder [62] was then run on the 13 selected *Serratia* spp. From this analysis, 1434 single-copy gene groups were identified and aligned using MAFFT [63], of which 643 were of suitable length for further analysis. The aligned single-copy gene groups were then tested for recombination using PhiTest via the Phipack package [64], with the window parameter set to 50 nucleotides. Of the groups tested, 13 showed recombination signals and were therefore omitted. The remaining nonrecombinant alignments were then concatenated, with a maximum likelihood tree then inferred using IQ_TREE 2 with the model Q.plant + F + I + R4 and 10,000 bootstraps [65].

FGD analysis was also undertaken which utilizes an ORFeome vs ORFeome analysis to cluster species by ORFeome similarities [24].

Average nucleotide identity scores (ANI’s) were calculated for each comparison between genome including 100% of the chromosomal sequence to determine percentage similarity using an ANI/AAI genome-based distance matrix calculator [66].

### Targeted mutagenesis of the itaconate region

The 3,677 bp *ita*-*ripC* amplicon generated using the primers ItaF and ItaR (Table 11) was digested with restriction enzyme XbaI and cloned into the analogous site of pUC19 [67] from where a tetracycline cassette was ligated into two Nco1 sites (deleting 547 bp to 792 bp of *ripC*) to form pUC19-RipC. The construct pUC19-RipC was then digested with EcoRI and the tetracycline tagged fragment then ligated into the analogous site of pJP5603 [68] to form pJPRipC. The sequence validated pJPRipC was then electroporated into *E. coli* ST18 [69] enabling its conjugation into the *S. entomophila* isolate 626 following the method of Martínez-García et al. [70]. Tetracycline-resistant transconjugants were patched on LB plates to determine pJP5603 encoded kanamycin sensitivity. Prospective recombinants were validated using the ItaF and ItaR primers (Table 11) and DNA sequencing of the resultant amplicon. The sequence validated *ripC* recombinant designated 626::RipC.

To construct pACRipC enabling the *trans* complement of 626::RipC, the *ripC* amplicon (Table 11) was digested with EcoRV and ligated into the analogous site of pACYC184 [71] to form 626::RipC (pACRipC). The sequence validated vector pACRipC_cm then electroporated into 626::RipC, to form 626::RipC (pACRipC).

### Bioassays

Field collected 3^rd^ instar *C. giveni* were pre-fed from where only healthy, feeding larvae were selected for bioassay assessments as outlined by Hurst et al. [9]. For maximum challenge bioassays the selected larvae were fed carrot (3–4 mm^3^ in size) inoculated via rolling on a bacterial lawn grown overnight on LB agar plates at 30 °C (approximately 1 × 10^8^ CFU per larvae). Each treatment comprised 12 larvae and was undertaken in duplicate. The treated carrot was administered on day zero, with fresh untreated carrot cubes provided on days three and six. Uninoculated carrot was used as the negative control and the positive controls comprised carrot cubes treated with either *S. entomophila* strain A1MO2 or *S. proteamaculans* AGR96X. Symptoms of disease (non-feeding, amber discolouration) were visually assessed on days three, six, nine, and 12.

LC_50_ was determined using the bioassay method but using different concentrations of the bacteria-derived from a serially diluted overnight culture (1 × 10^1^ to 1 × 10^4^ CFU/ml), where 5 µL of a dilution was pipetted onto a carrot cube.

For co-infection of *C. giveni* larvae a 50:50 infection was undertaken using a 3 mm^3^ cube of carrot inoculated with 5 µL of overnight culture (resulting in 3.1 × 10^7^ CFU 626 and 4.8 × 10^7^ CFU 626::RipC per carrot cube) that was fed to healthy grass grub larvae (~ 24 per treatment).

### Enumeration of bacteria from larval macerates

*Costelytra giveni* larvae were weighed before macerating in a total volume of 1 mL dd.H_2_0. Macerates of larvae removed at days 3, 6, 9, and 12 were subjected to serial dilution and plated onto CTA plates selective for wildtype *S. entomophila* 626 and the colonies then patched to LB agar tetracycline plates selective for 626::RipC. Three larval macerates were assessed at each time point. The isolates were validated as *S. entomophila* using media as outlined by O'Callaghan [16] and when required using genomic BOX-PCR DNA fingerprinting using the BOXA1R primer was used to validate *S. entomophila* isolates [72].

### Statistical analysis

*P*-values were generated using a two-sample t-test for bioassay data based on the instance of disease, death, or combined outcome relative to the untreated control for each assay using Minitab 18. Error bars used in graphs of bioassay data and bio infectivity assays generated in GraphPad Prism 9.2 were generated as the standard error of the mean.

## Supplementary Information

**Additional file 1.** COG breakdown of *Serratia entomophila *626 in comparison to the 5 selected *Serratia* species from Genbank. No. denotes number of COGs per isolate; % the percentage of the genome made up by this category.

## Data Availability

The datasets generated and/or analysed during the current study are available in the GenBank NIH genetic sequence database (GenBank: https://www.ncbi.nlm.nih.gov/genbank/) under the accession CP074347 (Table 2). Raw sequencing reads were deposited to the NCBI SRA archive under the accessions SRR19427101- SRR19427102.

## Abbreviations

Afp: Anti-feeding prophage
Amp: Ampicillin
ANI: Average nucleotide identity
BlastN: Nucleotide sequence/ query Blast search
BlastP: Protein sequence/ query Blast search
bp: Base pair
CFU: Colony-forming units
Cm: Chloramphenicol
COG: Cluster of Orthologous Group
DNA: Deoxyribonucleic acid
FGD: Functional genome distribution
HGT: Horizontal gene transfer
Kb: Kilobase
LB: Luria–Bertani growth medium
LT_50_: Lethal-time 50: minimum time to cause 50% death
Mb: Mega base
NCBI: National Center for Biotechnology Information
OD: Optical density
pADAP: Amber disease-associated plasmid
PBS: Phosphate-buffered saline
PCR: Polymerase chain reaction
RNA: Ribonucleic acid
rpm: Rotations per minute
Sep-TC: *Serratia entomophila* pathogenicity toxin complex
Tet: Tetracycline
tRNA: Transfer-ribonucleic acid
